# A Framework for the Monitoring and Evaluation of International Surgical Initiatives in Low- and Middle-Income Countries

**DOI:** 10.1371/journal.pone.0120368

**Published:** 2015-03-30

**Authors:** George M. Ibrahim, David W. Cadotte, Mark Bernstein

**Affiliations:** 1 Division of Neurosurgery, Department of Surgery, University of Toronto, Toronto, Ontario, Canada; 2 Institute of Medical Sciences, University of Toronto, Toronto, Ontario, Canada; UNAIDS, TRINIDAD AND TOBAGO

## Abstract

**Background:**

An estimated two billion people worldwide lack adequate access to surgical care. To address this humanitarian emergency, an increasing number of international surgical partnerships are emerging between developed and low- and middle-income countries (LMICs). At present, there are no clear indicators that may be used to assess the effectiveness of such initiatives.

**Study Design:**

We conducted an international qualitative study of 31 surgeons from developed and LMICs involved in international partnerships across a variety of subspecialties. Thematic analysis and grounded theory were applied in order to develop a practical framework that may be applied to monitor and evaluate global surgical initiatives.

**Results:**

Several themes emerged from the study: (i) there is a large unmet need to establish and maintain prospective databases in LMICs to inform the monitoring and evaluation of international surgical partnerships; (ii) assessment of initiatives must occur longitudinally over the span of several years; (ii) the domains of assessment are contextual and encompass cultural, institutional and regional factors; and (iv) evaluation strategies should explore broader impact within the community and country. Based on thematic analysis within the domains of inputs, outputs and outcomes, a framework for the monitoring and evaluation of international surgical initiatives, the Framework for the Assessment of InteRNational Surgical Success (FAIRNeSS) is proposed.

**Conclusions:**

In response to the increasing number of surgical partnerships between developed and LMICs, we propose a framework to monitor and evaluate international surgical initiatives.

## Introduction

An estimated two billion people worldwide lack adequate access to surgical care [1]. This humanitarian crisis is largely concentrated in low- and middle- income countries (LMICs), where inequities in access to surgery contribute to preventable death and disability [2]. Delayed or inadequate access to both general and sub-specialty surgical care is also associated with considerable psychosocial morbidity and stigma[3,4]. The role of surgery in the development of comprehensive healthcare systems and health promotion is therefore increasingly recognized. Importantly, surgical care is also increasingly understood to represent a cost-effective component of primary care in LMICs [5–7] and could play a critical role in improving multiple domains of healthcare [8]. Despite such recognition, surgical access remains poor and within many LMICs, the majority of surgical services are largely concentrated in urban centres, leaving rural communities with virtually no access to such care. In the resource-limited setting, a large proportion of the population may also be precluded from accessing surgical care due to a lack of capacity.

In response to the large unmet need for increased access to surgical care in LMICs, numerous organizations have begun to prioritize surgical capacity-building in the developing world[9]. For example, in 2009, a consensual partnership, the East African Neurosurgical Research Cooperative was formed in order to advance global health development in neurosurgery [10]. Additionally, individual surgeons have contributed their expertise to the amelioration of surgical care in LMICs through independent surgical missions [11–13]. Global surgery has also recently been emphasized as a means to achieve the Millennium Development Goals, with the formation of the World Health Organization (WHO) Global Initiative for Emergency and Essential Surgical Care (GIEESC) to strengthen surgical and anaesthetic services in LMICs [8,14].

With the global momentum to augment the response to surgical needs of LMICs through the establishment of collaborative partnerships with developed countries, it is increasingly important to provide accountability for activities to stakeholders, through effective monitoring and evaluation strategies. Such stakeholders may be funding sources in both developed and LMICs, local hospitals or multi-national organizations. Monitoring refers to routine tracking and reporting of priority information on a programme and its intended results, while evaluation is a process to determine their impact or value. These activities are important to secure continued funding for expanding surgical programmes in LMICs and to strengthen existing ones. Their importance is also buttressed by the widely recognized fact that well-intentioned medical missions abroad may have a paradoxical negative overall impact and are associated with important ethical challenges [15]. Such inadvertent effects are often the by-product of a lack of consideration of appropriateness, sustainability or comprehensiveness of the initiative.

The literature on international collaborations consists largely of opinion pieces [11] or single-centre observational [3] and philosophical work [15], with little analysis of relevant quality indicator or outcome measures. There is, therefore, an increasingly important need to develop consensus guidelines that can be readily applied to monitor and evaluate the impact of surgical partnerships. In the current study, we used qualitative research methodology and a grounded theory approach to develop a framework of standard indicators that may be used for the monitoring and evaluation of surgical partnerships between developed and LMICs. This work was inspired by existing monitoring and evaluation strategies for communicable diseases that are currently endorsed by supranational organizations [16]. Such a framework is intended to be applied in order to (a) formulate a monitoring and evaluation strategy by providing an overview of key issues to consider; (b) provide reports on the impact of initiatives during their implementation and scaling (i.e. to scale up); and (c) ensure quality control in the conduct of surgical programmes.

## Methods

### Study Design

The ethics board of the University Health Network (UHN) approved this study. Written consent was obtained from participants where feasible. If interviews were conducted via an online platform, verbal consent was obtained due to inability to obtain written documentation. Consent was recorded in all cases in the transcripts of the interviews performed. The REB approved the project and consent process. Qualitative case study methodology was employed with open-ended interviews with surgeons (consultants and residents) in developed and LMICs conducted face-to-face, as well as online via telecommunication. The set of semi-structured interview questions used as a nidus for discussion is presented in **S1 Table.** The questions were generated based on a logic model process [17,18]. This is a tool that is often used by program managers and evaluators to describe the effectiveness of programmes and describes logical linkages among program resources, activities, outputs and short-, intermediate-, and long-term outcomes. In circumstances where formal interviews were not feasible, the surgeons were asked to write a commentary to ascertain their thoughts on these questions. Where applicable, follow-up questions were asked to expand on the responses provided.

### Setting and Participants

Participants from developed and LMICs were invited to participate using a self-selected snowball method. The former were recruited based on their history of involvement in global surgery, evidenced by publications, membership in international surgical organizations (i.e. Foundation for International Education in Neurological Surgery; FIENS), and recommendations from colleagues.

Thirty-one interviews were conducted, which is approximately the number that conforms to typical sample sizes used in other qualitative research projects related to surgical patients and at which point saturation of themes is achieved [19]. Saturation is a term used in qualitative methodology to denote the point at which no new themes are expected to arise during subsequent interviews [20]. The interviews were audiotaped for subsequent transcription and analysis.

### Analysis

Analysis of the transcripts followed the techniques of data reduction, data display and drawing and verifying conclusions, all of which occurred concurrently [21]. The transcribed data were labelled descriptively, resulting in 42 categories during first-level coding. A sample of the analysis, with limited, de-identified transcripts containing representative information is included in **S2 Table**. A clear definition was allocated to each category, which was refined as categories were grouped by themes. Themes were subsequently parsed by evaluating redundancies and similarities. Connections between the themes were identified in order to establish links between them and develop conclusions.

## Results

### Participant Information

Thirty-one interviews were conducted. Thirteen interview were performed with surgeons from developed countries (DC-Surg) including Canada (n = 7), Norway (n = 1) and the United States (n = 5). These individuals have led surgical initiatives in numerous developing regions of the world, including Central and South America, Africa, Asia, Oceania and the Middle East. Eighteen interviews were conducted with surgeons from LMICs (LMIC-Surg), including Chile (n = 1), Ethiopia (n = 14), Kenya (n = 1), Nigeria (n = 1), Ukraine (n = 1). Saturation was reached, where new interviews did not contribute novel themes that did not emerge in prior interviews. The surgeons interviewed included general surgeons as well as sub-specialty surgeons (cardiac surgery, neurosurgery and urology).

### Thematic Analysis

Analysis of the interviews yielded five overarching themes that explain surgeons' perceptions of successful international surgical partnerships. Participants expressed a need to better contextually-relevant evidence to inform the monitoring and evaluation of missions, emphasized the importance of international collaboration, and the value of longitudinal, culturally-relevant and broad indicators to index the success of international missions. An analysis of the interviews is presented with direct quotations from participants.

(1) There is a large, unmet need to monitor and evaluate the success of missions through the establishment of prospective outcomes databases

All the surgeons interviewed unanimously expressed a strong opinion that the evaluation of surgical partnerships is of critical importance. It was found that the determination of quantifiable outcome indicators that can be used to index the success of surgical missions is a difficult, yet possible endeavour:

*“Really it is very difficult to assess the extent of our impact*. *When we do something*, *how far reaching is it*? *Geographically*, *does it stay in that hospital*? *Does it spread to the community*? *And in fact*, *in time—does it happen that day*? *Or is it sustained 3 months or 3 years from now*?” (DC-Surg)

*“I don’t even know how to assess capacity—and I’m fairly sure I’m not the only one*.” (LMIC-Surg)

A critical finding of the current study is that nearly all DC and LMIC surgeons stated that prospective data must be collected through comprehensive databases in order to adequately monitor and evaluate the success of international surgical partnerships:

*“If you don't know what you're measuring you can't fix it and that starts on an individual level*.” (DC-Surg)

*“There are some surgeries done*, *but we don't measure anything*…*you have to measure outcomes*, *that's the only way I know if you're well-trained or not*.” (LMIC-Surg)

While all the interviews endorsed the concept of monitoring complications and outcomes, as well as the number and complexity of cases being performed, some went so far as to suggest that databases should include measures of patient satisfaction and quality of care delivery:

*“Patient satisfaction should be monitored*. *For example*, *the relatives*, *what were they expecting*, *what did they see and what they are feeling*.” (LMIC-Surg)

Many interviewees acknowledged the monumental effort required to establish such databases. There was also much optimism amongst LMIC-Surg that this was possible, and indeed, numerous international partnerships have already started collected prospective data:

*“We are starting a database—it is one year old and we log the trauma cases and elective cases*… *we are focusing on peri-operative outcomes*…*we record all that—so how many mortalities we have and what are the main causes of mortality*…*So this will be periodically followed up—like monthly or every three months we have a summary of all the activities and we discuss this—at least we have started this*.” (LMIC-Surg)

The benefits of establishing a database are far reaching. Many interviewees emphasized the importance of knowing the prevalence of diseases and institutional complication rates in order to mitigate their impact. Others also viewed prospective databases as an effective means to identify deficiency in care that may be amenable to educational intervention or to lobby governments and funding agencies for change:

“Data—well, the best is to collect data on the number of patients operated, the number of outpatients that are seen, mortality… that can be compiled into a book and presented to the government. And we cannot only limit our self to aid facilities—we need the government to contribute.” (LMIC-Surg)

*“We initiated [log books]*…*Now they have a framework*…*to design a curriculum based on what their needs are*.” (DC-Surg)

(2) There is a greater need to coordinate international initiatives within the larger global surgical community

It also emerged that the success of a surgical mission is often determined by the planning that takes place before the team arrives at the local centre:

*“Generally*… *things click well between visitor and visitee*, *particularly if a lot of homework is done in advance*” (DC-Surg)

*“It is good to collect all the cases and discuss with them before they come*. *It might be better for me to collect cases here and then get exposure”* (LMIC-Surg)

There was also a common concern regarding the lack of coordination of international initiatives within the larger global surgical community:

*“There [are] a lot of people coming out of the woodwork just dropping in and doing things*. *Absolutely—there should be better coordination*.” (DC-Surg)

*“Some of the time there are two or more [international visitors] at the same time*. *But it would be good if there was a schedule*. *If we know ahead of time and discuss things it would be good*.” (LMIC-Surg)

(3) Monitoring and evaluation must be performed longitudinally over the span of many years

Nearly all surgeons emphasized the importance of a long-term commitment and the fact that it is impossible to gauge the success of surgical mission in the short term:

“*I simply think that you cannot achieve any measurable goals in 6 months to 1 year—you can achieve something in 5 years*” (DC-Surg)

“*It's not worth to come back for one second and say a few things and leave*. *The person has to come on a slightly regular basis*, *maybe once a year*.” (LMIC-Surg)

Many DC-Surg felt that an ongoing collaboration is the ideal means by which to achieve the greatest impact in LMICs:

*“The short term goals are to create a collaboration with the group to develop a rapport so it's fertile ground for further teaching So that's the short term plan*, *which melds into the long term plan*. *Are you we going to have a future relationship*?” (DC-Surg)

*“A long term engagement and addressing all the issues—with commitment and follow through—this is the only way to make it work*.” (DC-Surg)

As part of a longitudinal relationship, many surgeons both in developed and LMICs recognized the importance of reciprocal visits and of having LMIC-Surg observe surgical units in developed countries:

*“I bring fellows here or observers here or students here from overseas*, *one of the most important things*…*is watching how our system works*. *How respectfully patients are treated*, *how the system works on behalf of the patient and how patients care and lobby for us to do a good job*, *even in subtle ways in the clinic setting*.” (DC-Surg)

*“We learn a lot from the way you communicate with your consultants*.” (LMIC-Surg)

While the majority of those interviewed emphasized the importance of cultural sensitivity and respect for the local traditions, nearly all surgeons, both from developed and LMICs stressed the importance of changing medical and surgical culture in LMICs:

*“The expectations of people*…*are not that high and therefore the expectations of the surgeons on themselves are not that high*. *One of the problems that I see frequently is a sense of resignation*.” (DC-Surg)

*“The reason why we didn’t have training*…*is because of laziness*. *I believe that—the training is intense [in developed countries]—here it is not*.” (LMIC-Surg)

Changes to medical culture encompassed several domains. First, it was felt that there was a need to emphasize personal initiative, and responsibility in the surgical culture in LMICs:

*“The whole idea of personal responsibility*, *reliability—professionalism—is not being taught there*.” (DC-Surg)

*“One is the feeling of responsibility—which means accountability*....*If I’m on duty and I don’t come in and a patient dies*, *nobody asks me*. *Unless my conscience asks me*, *it's very unlikely anyone else will*.” (LMIC-Surg)

Second, there was a general consensus that mistakes must be acknowledged, reviewed and mitigated:

*“They tend to burry their mistakes*. *I tried to get an M&M [session] once*… *How can you learn if you don’t analyze in a really critical way your mistakes*? *They don’t do that*.” (DC-Surg)

“Maybe one thing we just have to once in a while sit down and discuss things…and what needs to be changed.” (LMIC-Surg)

Third, it was felt that greater advocacy is needed for patients:

*“I am not resigning myself to the fact that this patient is going to be cancelled for the 3* ^*rd*^ *week—I am going to lobby and get him into the OR tomorrow*…*right now*, *you don’t get the feeling that anybody cares very much if a case gets cancelled*.” (DC-Surg)

Indicators of such changes to medical and surgical culture included the academic productivity of the departments, increased funding from government, having surgeons stay in publically-funded hospitals and greater notoriety for the institution:

*“Research is one way to build clout*, *which would allow them to advocate for further funding and advance their clinical practice*.” (DC-Surg)

*“In the same way try to convince them to stay in their centre*, *because we have a private system which is very very strong*, *which tries to take away people from the academic centres*.” (LMIC-Surg)

It was felt that changes to medical and surgical culture need to be complemented by competency-based training and relevant examinations:

*“We should somehow work out the number of procedures a resident should do—a log book*. *And unless he has done a certain number of procedures he can’t graduate”* (LMIC-Surg)

(4) Monitoring and evaluation strategies should include broader regional and national indicators

It was widely accepted that monitoring and evaluation strategies must also assess the regional and national issues relevant to the practice of surgery:

*“When we go*, *we don't just go to help the patients*, *we go to help the society*.” (DC-Surg)

*“We have to change the attitude of the governments*, *especially with regard to financial and you have to know that [surgical] problems are not limited—it is very vast—especially in our country”* (LMIC-Surg)

There are several ways in which the impact of surgical initiatives on regional and national levels may be assessed. First, it was felt that collaboration between large referral centres performing surgery within a specific LMIC is an important measure of success:

*“If we achieve to get collaboration between centres that would be a massive success*…*That would mean we are all working for one thing*. *That would be a big change in concept and attitude and trying to work together*.” (LMIC-Surg)

Second, it was important to increased awareness and prevention of surgical diseases in the wider medical community:

*“Awareness of neurosurgery by other health professionals is not good—they think that neurosurgical outcomes are so bad that it is not worth intervening*.” (LMIC-Surg)

Third, socioeconomic indicators, including more rehabilitation facilities, fewer patients being treated abroad, more efficient healthcare spending and improved transportation and triage systems were deemed important considerations:

*“If [LMIC] needs to spend 48 billion next year in health care instead of 72 billion*, *that would a tremendous outcome measure*.” (DC-Surg)

*“You can look at how many people used to go for surgery abroad from [LMIC]—and track that after five years*.” (LMIC-Surg)

*“People present to the emergency room with very advanced states of disease*, *they get admitted to hospital*, *they wait for weeks to months for surgery*.” (DC-Surg)

## Discussion

With growing recognition of the global burden of surgical disease, an increasing number of surgical partnerships between developed and LMICs are emerging. The current report used qualitative research methodology to develop a framework to monitor and evaluate such initiatives. Several critical themes emerged in interviews. First, we identify a large, unmet need to maintain prospective databases of patient outcomes in LMICs. Second, our study found that international initiatives should be better coordinated within the global surgical community. Finally, we report salient cultural, institutional and regional indicators that should be considered when monitoring and evaluating surgical programmes in LMICs.

**Fig 1:**
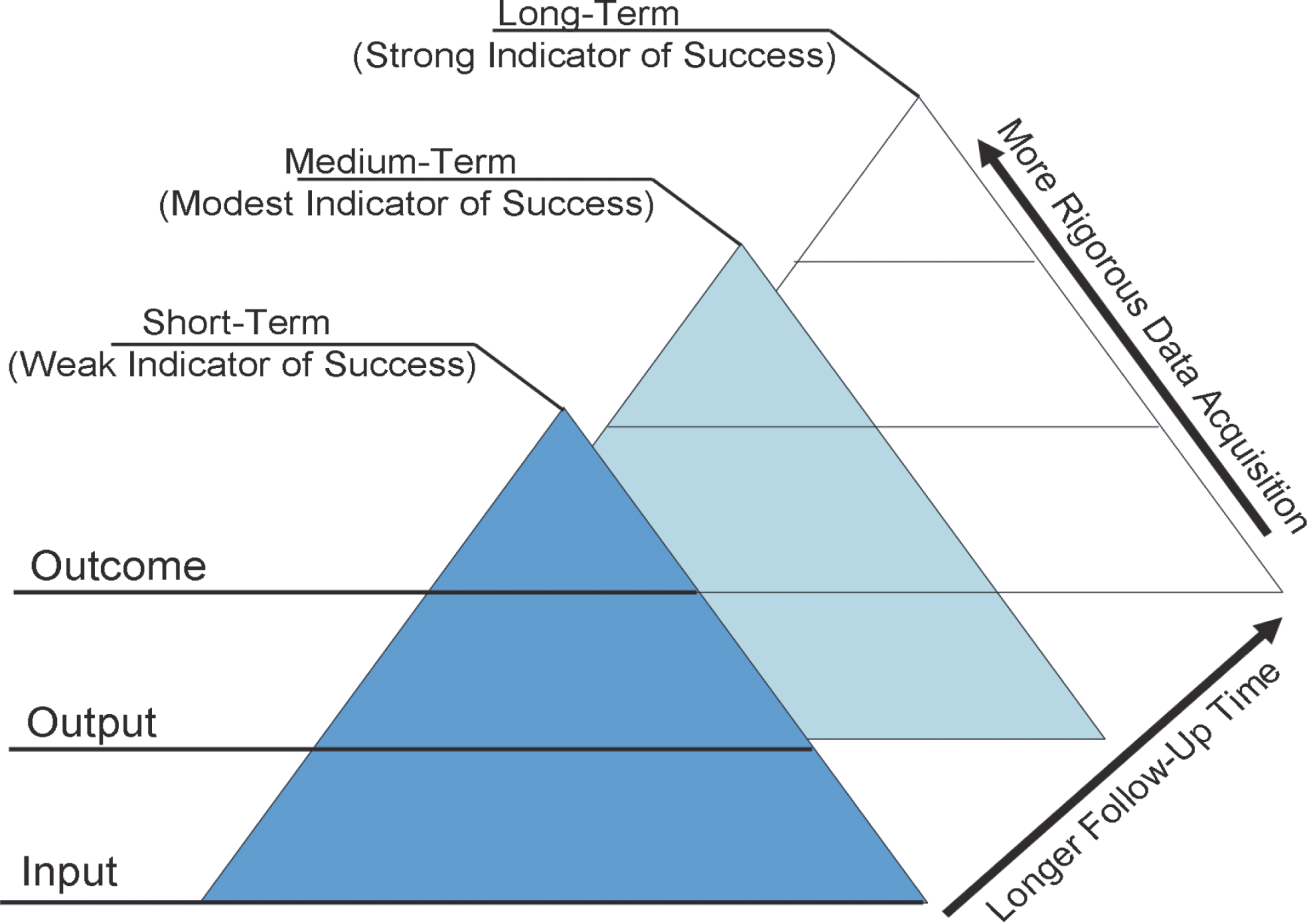
Logic model of program monitoring and evaluation. Inputs, outputs and outcomes of programmes are evaluated with increasingly rigorous data collection. Inputs refer to equipment, finances and time invested in order to achieve outputs, such as better trained surgeons or increased surgical output. The outputs result in processes, such as ongoing staff training. The outputs, if successful lead to outcomes that have a measureable impact, such as lower morbidity and mortality of surgical interventions in LMICs. Success of the program is evaluated over the short, medium and long term whereby short term indicators of success, such as the number of operations performed during a single visit, offer a weak indication of success; Long term indicators of success, such as a recorded decrease in morbidity associated with a particular surgical condition, offer a strong indicator of program success.

There are various frameworks that may be applied to the synthesis of monitoring and evaluation indicators. Using thematic analysis and a grounded theory approach, we applied the input-process-output-outcome-impact (logic model[22]) framework to organize germane indicators of successful surgical programmes into the FAIRNeSS criteria (The Framework for the Assessment of InteRNational Surgical Success; **Table 1**). Inputs refer to equipment, finances and time invested in order to achieve outputs, such as better trained surgeons or increased surgical output. The outputs result in processes, such as ongoing staff training. The outputs, if successful lead to outcomes that have a measureable impact, such as lower morbidity and mortality of surgical interventions in LMICs. Measurement of growing impact (from inputs to outputs and outcomes) requires increasingly rigorous data acquisition at ongoing intervals (**Fig. 1**). Success of the program is evaluated over the short, medium and long term whereby short term indicators of success, such as the number of operations performed during a single visit, offer a weak indication of success. Long term indicators of success, such as a recorded decrease in morbidity associated with a particular surgical condition, offer a strong indicator of program success.

**Table 1:** The Framework for the Assessment of InteRNational Surgical Success (FAIRNeSS) Criteria.

	Category	Item	No	Some-what	Yes
**Short-Term Indicators (If achieved, these offer a *weak* indicator of success)**					
	Input				
		The visit was coordinated with the international community beforehand			
		Upon arrival, cases were ready to review and operate			
		Equipment/resources (including books) brought was appropriate for setting			
		Resources and training for allied health team (nurses, physiotherapists, etc) were considered			
		The local team was introduced to data collection strategies and appropriate research methods			
	Output				
		The number of operative cases was acceptable			
		Local team satisfied with intra- and extra-operative teaching			
		The visiting team and local surgeons **together** met with regional leaders, politicians or healthcare decision-makers			
		The visit generated publicity for the local centre			
**Intermediate-Term Indicators (If achieved, these offer a *modest* indicator of success)**					
	Input				
		The resources (equipment and training material) brought continues to be used			
		The local team is able to troubleshoot equipment failure without outside assistance			
	Output				
		A prospective database has been established for recording of cases and complications			
		The partnership has created incentive to retain current staff and trainees at the local centre			
		The local team continues teaching rounds and presentations independently			
		Morbidity and mortality is recorded and discussed			
		The allied health team continues to participate in teaching rounds			
		Local centre has training facilities, such as cadaveric or surgical-skills labs.			
		The local team is publishing peer-reviewed papers			
		Rehabilitation centres are available for patients			
	Outcome				
		An ongoing relationship is established with local surgeons			
		Training surgeons undergo formal evaluations			
		The local surgeons can better utilize their available resources (i.e. ICU beds, ventilators, etc)			
		Regional leaders prioritize surgical care and financially support surgeons in public institutions			
**Long-Term Indicators (If achieved, these offer a *strong* indicator of success)**					
	Input				
		The local team has secured their own source of equipment without outside assistance			
	Output				
		More surgeons are operating in the country			
		surgery is available throughout the country			
		Centres are operating on greater volumes			
		More complex cases are being performed			
		Better facilities (including intensive care and imaging are available)			
	Outcome				
		Decreased peri-operative morbidity and complications			
		Measurable improvements in patient quality-of-life			
		Ethical considerations included in decision-making			
		Appropriate prevention, referral and triage systems are in place			
		Surgeons are staying in the country after training			
		Fewer patients are going abroad for surgical care			
		Junior surgeons are involved in teaching and training			
		The country/region has a surgical or professional regulatory society			
		The centre is recognized regionally and internationally			

The FAIRNeSS framework does not aim to provide an all-encompassing overview of all possible indicators. Rather it provides surgeons, organizations and other parties with a set of common indicators that have been gathered through our qualitative methodology from stakeholders in both developed and LMICs. In this way, we propose a formalized set of indicators that may be used for monitoring and evaluation strategies, a subset of which have been previously applied to the same end [13,23]. Such a common and comprehensive monitoring and evaluation framework is advantageous for several reasons. It contributes to efficient usage of data and resources by ensuring indicators and sampling methodologies are comparable over time. Furthermore, data generated from the FAIRNeSS monitoring and evaluation strategy may serve the needs of numerous stakeholders, including program managers, researchers or philanthropists. Finally, we provide the best available set of common indicators that may be used to measure sustainable impact of surgical initiatives in LMICs.

**Fig 2:**
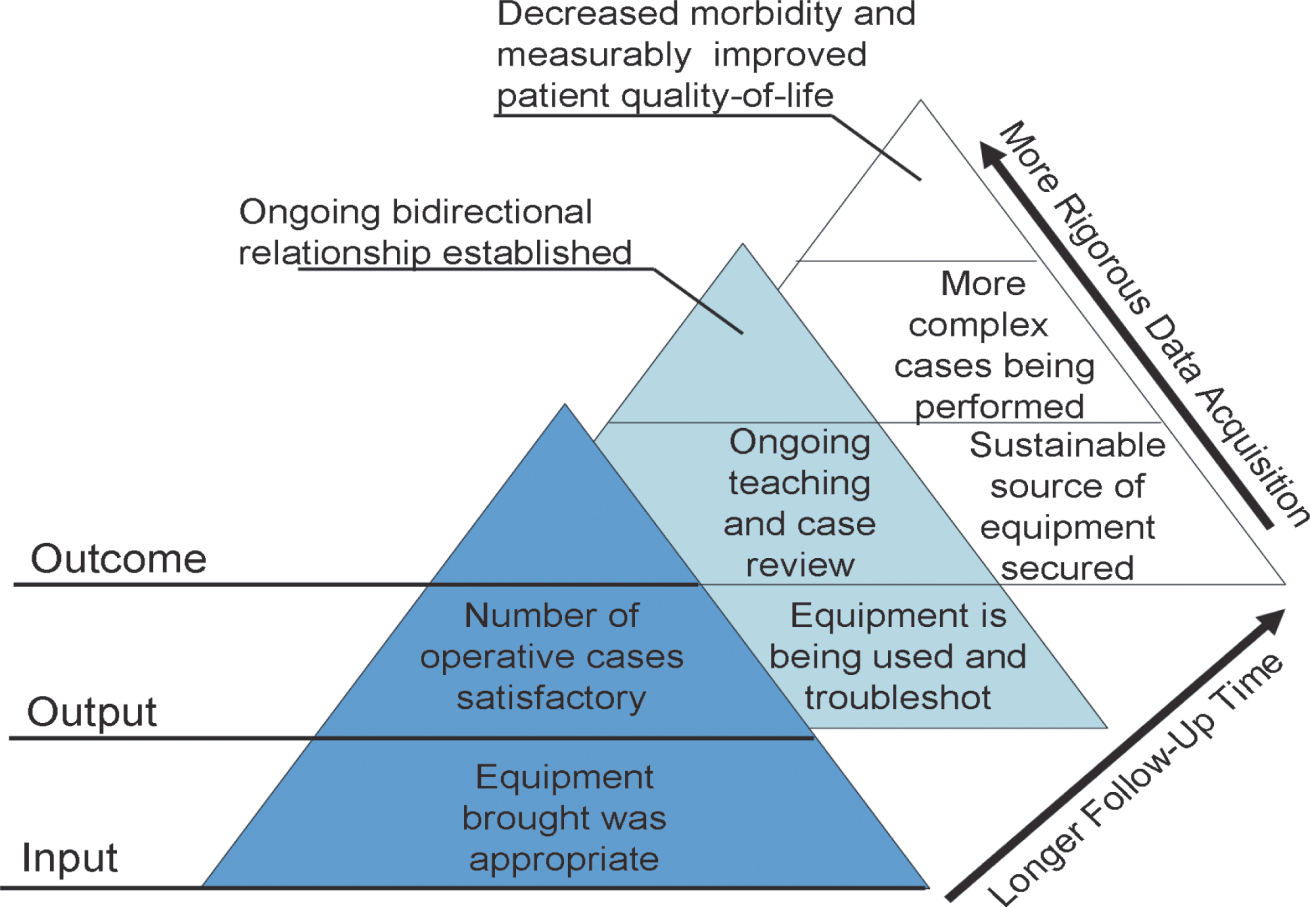
Longitudinal and hierarchical organization of the FAIRNESS framework. The FAIRNESS framework encompasses indicators that are increasingly acquired through rigorous data collection as well as with longer follow-up intervals.

Assessment of the success of a surgical mission cannot be accomplished without the dimension of time, in order to effectively assess the long-term impact of interventions on local populations. Indeed, formal partnerships between developed and LMICs are often the end result of multiple incremental steps and collaborations, which must be evaluated on different temporal scales. We propose that the different elements of the framework be applied to the monitoring and evaluation of surgical programmes at differing intervals (**Table 2**). An example of indicators assessed across the longitudinal (increasing value of longer follow-up) and hierarchical (increasing impact of inputs-outputs-outcomes) dimensions is shown in **Fig. 2**

**Table 2:** Types of indicators and recommended frequency of measurement*.

Type of Indicator	Frequency of Measurement
Input	Continuously
Output	Quarterly, semi-annually or annually
Outcome	2 to 5 years

*Adapted from Monitoring and Evaluation Toolkit for communicable diseases ^16^

In the current report, we have not attempted to quantify what may be deemed an acceptable target to fulfill the criteria of each indicator. Our purpose primary was to establish a set of common indicators based on the opinions of surgeons from developed and LMICs that are important to the monitoring and evaluation of surgical initiatives. What is deemed adequate to fulfill each indicator may differ amongst different locations and with longitudinal follow-up. The FAIRNeSS criteria may therefore be used as nidus for the development of region- and center-specific baselines and targets, which could be quantified through ongoing field work.

There are several limitations to our study. First, the generalizability of our results has not been validated prospectively. While the objective of qualitative analysis is not necessarily to provide generalizable results, we did find remarkable similarity in responses from surgeons in both the developed and LMICs. Furthermore, we chose to exclusively interview surgeons, since they have historically led international surgical initiatives in LMICs. Future studies may aim to refine or add to our findings by including the perspective of other individuals, such as hospital administrators, regional politicians and decision-makers. There were also a disproportionate number of respondents from a single LMIC, Ethiopia, and the majority of surgeons from the developed world were North American. Despite this, we found consistent responses across the interviewees irrespective of geography, specialties and among surgeons from LMICs and developing countries. Whether such findings would be similar across other settings remains to be elucidated. Furthermore, while it is possible that important themes were overlooked, saturation was achieved, whereby additional interviews did not contribute novel themes. It is important to emphasize that the FAIRNeSS monitoring and evaluation strategy is a framework that is subject to constant re-evaluation and change depending on specific national or program goals. When possible, outputs and outcomes should strive to contain a local, culturally meaningful context. In this respect, future investigators will most assuredly contribute additional measures that take into account a vast cultural diversity between LMICs that are undertaking steps to improve surgical capacity. The present framework is an important set of common indicators for use by the international surgical community in monitoring and evaluating surgical partnerships.

## Conclusions

Using qualitative research methodology, we performed an international study of surgeons from developed and LMICs to order to create the FAIRNeSS framework for the evaluation and monitoring of international surgical initiatives. Based on these results, we can make several suggestions to improve the probability of success of such missions: (1) Databases must be established to record and monitor patient outcomes; (2) Greater collaboration between international surgery stakeholders is required to maximize the benefit of surgical initiatives; and (3) Cultural, institutional and regional indicators must be considered when monitoring and evaluating programmes. Given the growing global response to the need for greater surgical capacity in LMICs, the FAIRNeSS framework may represent a useful tool to assess the success of surgical initiatives.

## Supporting Information

S1 Table Semi-structured questions to surgeons from developed and LMICs. (DOC)

S2 Table Sample analysis of interview transcripts. (DOC)
