# Supplementary material for: A Framework for the Monitoring and Evaluation of International Surgical Initiatives in Low- and Middle-Income Countries
Source: PLoS One. 2015 Mar 30;10(3):e0120368. doi: 10.1371/journal.pone.0120368 (PMC4379101; doi:10.1371/journal.pone.0120368)
Supplement: S1 Table — (DOC) [file pone.0120368.s001.doc]

**Supplementary Table S1: Semi-structured questions to surgeons from developed and LMICs**

| **Interview with Neurosurgeons from Developed Countries** | |
| --- | --- |
|  | 1. How can neurosurgical care be improved in LMICs? |
|  | 2. How do you think partnerships between developed/LMIC neurosurgeons will impact neurosurgical care in LMICs? |
|  | 3. What short term goals do you have? |
|  | 4. What long term goals do you have? |
|  | 5. Do you expect the benefits of collaborations to remain local or extend throughout the country? |
|  | 6. What will it look like when there is optimal neurosurgical care in LMICs? |
|  | 7. What are possible indicators of an optimal situation? |
|  | 8. How can data be collected to monitor this? |
|  | 9. What behaviours need to change to achieve optimal delivery of neurosurgical care in LMICs? |
|  | 10. What problems have you encountered in assessing neurosurgical capacity? |
|  | 11. How would you suggest that neurosurgical capacity be assessed in the long term? |
|  | 12. What specific resources are required to improve delivery of neurosurgical care in LMICs? |
|  | 13. What knowledge or skills do people need to learn when neurosurgeons from developed countries (and their teams, if applicable) visit LMICs in order to improve the delivery of neurosurgical care there? |
| **Interview with Neurosurgeons from LMICs** | |
|  | 1. How can neurosurgical care be improved in LMICs? |
|  | 2. How do you think partnerships between developed/LMICs neurosurgeons will impact neurosurgical care in LMICs? |
|  | 3. What short term goals do you have? |
|  | 4. What long term goals do you have? |
|  | 5. Do you expect the benefits of collaborations to remain local or extend throughout the country? |
|  | 6. What will it look like when there is optimal neurosurgical care in LMICs? |
|  | 7. What are possible indicators of an optimal situation? |
|  | 8. How can data be collected to monitor this? |
|  | 9. What behaviours need to change to achieve optimal delivery of neurosurgical care in LMICs? |
|  | 10. What problems have you encountered in assessing neurosurgical capacity? |
|  | 11. How would you suggest that neurosurgical capacity be assessed in the long term? |
|  | 12. What knowledge or skills do people need to learn when neurosurgeons from developed countries (and their teams, if applicable) visit LMICs in order to improve the delivery of neurosurgical care there? |
|  | 13. What specific resources are required to improve the delivery of care in LMICs? |
