# Supplementary material for: A Framework for the Monitoring and Evaluation of International Surgical Initiatives in Low- and Middle-Income Countries
Source: PLoS One. 2015 Mar 30;10(3):e0120368. doi: 10.1371/journal.pone.0120368 (PMC4379101; doi:10.1371/journal.pone.0120368)
Supplement: S2 Table — (DOC) [file pone.0120368.s002.doc]

Supplementary Table S2: Sample Analysis of Interview Transcripts

| Outcome |  |  |  |  |
| --- | --- | --- | --- | --- |
|  | Institutional |  |  |  |
|  |  | Changes in medical culture |  |  |
|  |  |  | Professionalism | - Instilling initiative, professionalism, and enthusiasm: "“We don’t do it.' 'But you have the equipment' 'We don’t do it' They have to do that" - I don't think they have the luxury of being able to deliberate and think about problems the way we do and the way we are able to teach our residents. So they are not as good critical thinkers. - they have to be a little more driven, a little more desirous, a little more focused and a little bit more intense about being as good as they can be. - where they see how work, how we think, how we are driven – these are personality traits that they can learn from - neurosurgery is at the bottom of the barrel – they are not respected, at least they do not see themselves as anywhere near the head of the pack in terms of leadership, medical capabilities, and so forth. - it is clear if someone is not behaving in a professional, responsible manner. That never seems to get addressed there. - Maybe more dedication – there is still room for more dedication - cultivating [younger trainees]ethically – and also trying to cultivate the feeling of responsibility. - back home you have to understand that people don't believe very much in research and in goals |
|  |  |  | Ownership / Responsibility of Patients | - they have to have a culture that they don’t have: of, this guy is sick – and we’re going to take care of him" / " is a change in the approach to health care – the approach to patient care. They don’t do it. - everyone must know who is responsible for a specific task or a specific patient... They don’t want to take individual responsibility.... It requires you have someone to write this out – it requires leadership. - I think having strong leadership is a critical thing. Not just having a plan for everything and everybody – but from a role model perspective - So as mentioned, the staff, the people responsible for doing neurosurgery have to get a bit more serious about it. - Things like judgment, maintenance, and infrastructure – are not priorities in the culture. - The whole idea of personal responsibility, reliability – professionalism – that is not being taught there. - too much emphasis has gone into just operating / surgical skills. Professionalism in the sense of an ethical neurosurgical practice – and the integrity that goes along with it. - They are always late, disappear in the middle of the day. They go and do private cases. - One is the feeling of responsibility – which means accountability. Now – if I’m on duty – and I don’t come in – and a patient dies – nobody asks me. Unless my conscious asks me – its very unlikely anyone else will... If I am to take on all of the accountability – then I have to be incentivized for it. So, to change the culture – you have to match accountability with incentive. Both the carrot and the stick have to be there. - Dedication to profession is totally lacking in Ethiopia as is good leadership. - our character – everybody is negligent – if you are persistent, if you see our character – you will automatically quit every activity that you bring to this country. - it’s not that I don’t want to have neurosurgery. No one has an answer why we are like that, why is everyone positive – no purpose to what we are doing – but things go slow. - you need responsibility, sympathy for the patient – he should be honest. If he loses these things he cannot practice medicine. |
|  |  |  | Acknowledgement of mistakes | - They tend to burry their mistakes. I tried to get a M&M once... How can you learn if you don’t analyze in a really critical way your mistakes. They don’t do that. - I think part of is has to be cultural – they just don’t correct each other. - Maybe one thing we just have to once and a while sit down and discuss things – in the past few months or so – and what needs to be changes. And I think our practice needs to be uniform – so sessions to discuss things together. |
|  |  |  | Advocacy | - I am not reigning myself to the fact that this patient is going to be cancelled for the 3rd week – I am going to lobby and get him into the OR tomorrow. I think a little bit of patient centered advocacy type of approach by the surgeons – right now, you don’t get the feeling that anybody cares very much if a case gets cancelled – it just means that they can go home a little early or go to their private practice earlier. That is a very pervasive thing – and the job ain’t going to get done with that kind of culture. - They need to dress better than they dress, they need to act better than they act – they need to be more involved politically in the surgery department, in the hospital – they need to conduct themselves professionally - No. No. No, our culture is very similar to western culture. No. |
|  | Regional / National |  |  |  |
|  |  | Shifting Demographics |  |  |
|  |  |  | Medical tourism | - a lot of the people who are wealthy, who can afford to go outside to south aftica or wherever – if you could measure this – and measure this in 5-10 years later and demonstrate that this has gone down somewhat – then you can make an inference that the system has improved markedly. - Now, ACDF people are going out of the country. Why don’t we have a routine practice here? - It is unfair to send patients to India, to Thailand or to see them dying at home... When they go out – even if they can afford it – they money they are taking is a lot. - You can look at how many people used to go for surgery abroad from Ethiopia – and track that after five years. |
|  |  | Policy Changes |  |  |
|  |  |  | Recognition and emphasis on surgical care. | - the ministry of health is interested in infectious disease, HIV, TB and malaria" - They think that our only problem is malaria, TB, they say my problem is maternal death |
|  |  |  | Public funding of neurosurgery | - they’re going to build a world class hospital – with venture capital by the way – so I get up and I say ‘but how is this going to affect the health care of the population' - but I think we have to talk about the many millions more people with no money at all |
|  |  |  | Support for neurosurgeons | - when they go home, they have to be supported - the government has to pitch in and pay them a living wage so they can concentrate on their university responsibilities |
|  |  | Regulation | Regulation of Neurosurgery | - and government or the college can give a surgical certificate – to control the training and give the license |
|  |  | Infrastructure Changes |  |  |
|  |  |  | Rehabilitation Centres | - he’s never going to get out of bed because they don’t have any rehabilitation facilities |
|  |  |  | Investments in triage and transportation | - transportation. Some of these people undergo significant delays to care - there is no national ambulance system to bring them in. What? On busses? Taxis? This is a big public health issue that comes down to finances and money. |
|  |  |  | Organization | - but they need to fix their systems – to develop their systems of same day admission programs, pre-admission, organized imaging, etc. Now – it is chaotic – people present to the emergency room with very advanced states of disease, they get admitted to hospital, they wait for weeks to months for surgery |
|  |  | Prevention | Prevention | - we are covering more trauma patients and pediatrics and infectious diseases so in the long term at least we can work more to prevent those things and second to just see the patterns of diseases like myelomeningocele and hydrocephalus and to provide the best service. |
|  |  | Primary care education |  |  |
|  |  |  | Referral of cases | - They don’t know what they are looking at. They don’t have appropriate referral mechanisms |
|  |  | Expansion |  |  |
|  |  |  | Increased number of centers | - One neurosurgeon can’t sustain a practice - establish independent neurosurgical teaching units throughout the country - we need a new neurosurgical center which we can do vascular, advanced neurosurgery, even if we want to have a gamma knife they have to have a single center. |
